# COVID-19 Vaccination Does Not Affect Reproductive Health Parameters in Men

**DOI:** 10.3389/fpubh.2022.839967

**Published:** 2022-02-02

**Authors:** Marco Reschini, Luca Pagliardini, Luca Boeri, Francesca Piazzini, Veronica Bandini, Gianfranco Fornelli, Carolina Dolci, Greta Chiara Cermisoni, Paola Viganò, Edgardo Somigliana, Maria Elisabetta Coccia, Enrico Papaleo

**Affiliations:** ^1^Infertility Unit, Fondazione IRCCS Ca' Granda Ospedale Maggiore Policlinico, Milan, Italy; ^2^Reproductive Sciences Laboratory, Obstetrics and Gynecology Unit, IRCCS San Raffaele Scientific Institute, Milan, Italy; ^3^Centro Scienze Natalità, Obstetrics and Gynecology Unit, IRCCS San Raffaele Scientific Institute, Milan, Italy; ^4^Urology Unit, Fondazione IRCCS Ca' Granda Ospedale Maggiore Policlinico, Milan, Italy; ^5^Department of Clinical Sciences and Community Health, Università degli Studi di Milano, Milan, Italy; ^6^Assisted Reproductive Unit–Careggi Hospital–University of Florence, Florence, Italy

**Keywords:** reproduction, COVID-19 vaccine, infertility, fertilization rate, semen, sperm, fertilization

## Abstract

With the implementation of COVID-19 vaccine up-take, doubts regarding the impact of immunization on future fertility have begun to emerge. We have examined vaccine safety on male reproductive health. We set up a multicentre (three infertility centers), retrospective study in order to assess semen parameters and fertilization rate of one hundred-six men in a pairwise comparison between the first and second assisted reproduction technology (ART) attempt, performed respectively before and after COVID-19 vaccination. Median time (range) between the first vaccine dose and the second ART cycle was 75 days (39–112). Semen parameters did not change before and after the exposure. Fertilization rate was also similar before and after vaccination. Twenty-five patients (24%) were oligozoospermic before the vaccination while 26 (25%) after the exposure (*P* = 0.87). Severe asthenozoospermia were present in 11 patients before as well as after the exposure. No difference was observed even after considering different types of vaccines (mRNA or viral vector). COVID-19 vaccination did not affect sperm quality and fertilization capacity of men undergoing ART treatments and should be considered safe for men's reproductive health.

## Introduction

Both types of COVID-10 vaccines, the messenger RNA (mRNA) vaccines and the vaccines utilizing a viral vector, have been shown to reduce COVID-19 infections, transmissions, hospitalizations and deaths in randomized controlled trials and real-world effectiveness studies (1). Evidence of the short- to medium-term safety of these vaccines is accumulating. Besides the common and usually mild side effects, such as the low-grade fever and the pain at the injection site, some major, but thankfully, uncommon, adverse reactions have been reported during the post marketing surveillance phase (2–4). The identification of other adverse events is now a global scientific priority.

Despite the high efficacy found in clinical trials, a sizeable minority of people in civilized countries does not plan to get a COVID-19 vaccine. The speed and urgency at which the vaccines were initially created and authorized caused some concern. With the implementation of the vaccine up-take, questions regarding the impact of the vaccine on future reproductive health have begun to emerge. Headlines have appeared across multiple social media platforms questioning the effects of the newly authorized vaccines on fertility, with little or no scientific evidence supporting the claims. In this regard, recent studies have shown that both BNT162b2 and mRNA-1273 vaccinations have no influence on sperm parameters of 45 young volunteers (5, 6). However, the impact of the vaccine on gamete functional competence has not been assessed. Moreover, in this selected young population, the overall presence of semen parameters within the normal ranges may have hidden subtle differences potentially attributed to the vaccine. Therefore, in a larger sample of patients undergoing infertility treatments for pregnancy seeking, we have evaluated semen parameters in a pairwise comparison between the first and second assisted reproduction technology (ART) attempt, performed respectively before and after COVID-19 vaccination. Fertilization rate as an indicator of sperm developmental competence has also been measured before and after the exposition to the vaccine (7). The potential effects of the two different vaccine-types were also considered.

## Methods

### Study Design

The study design was developed to examine vaccine safety. We compared (for each case) the parameters of interest after the exposure (after vaccination) with those observed in the unexposed period (baseline). The study was restricted to men who met the following eligibility criteria: (i) age > 18 years; (ii) have undergone two cycles of intrauterine insemination (IUI) or *in vitro* fertilization (conventional IVF or ICSI) before and after vaccination in the context of the couple's infertility management; (iii) evaluation of basal semen parameters before and after the exposure in the context of the infertility management. Those with COVID-19 symptoms or a positive test result within 90 days were excluded. None of the patients received any fertility-based medical treatments or surgical interventions between the unexposed and exposed periods. Men provided semen samples after 2 to 4 days of abstinence. IUI and IVF were standardized and performed as previously described (8–10). For IVF, oocyte collection was performed 36 h after triggering of ovulation. After 2–3 h incubation, oocytes were allocated to conventional *in vitro* fertilization or ICSI based on the semen characteristics. For ICSI, denudation of the cumulus oophorus was performed as previously described (11). Inseminated or injected oocyte were cultured in microdrops of specific medium under oil. Sixteen-eighteen hours after insemination or ICSI, all oocytes were checked for fertilization (two pronuclei) as previously described (12).

### Outcome Measures

Semen evaluation was performed before and after vaccination on the days of oocyte retrieval for IVF or on the same days of IUI. The analysis was done in the andrology laboratory, located nearby the embryology laboratory by trained embryologists. All semen parameters were assessed according to the 2010 World Health Organization (WHO) guideline laboratory manual for the examination and processing of human semen as previously described (13). The following variables were taken into consideration: volume (mL), sperm concentration (Number/mL), motility (%) and morphology (%). Sperm motility was graded into total (progressive + non-progressive motility) and progressive motility. Total sperm count (volume × sperm concentration) and total number of progressively motile sperm (%) were also calculated. Both internal and external quality control programmes have been established in the laboratories in order to control random and systematic errors and interlaboratory differences. Fertilization rate was calculated by dividing the number of fertilized oocytes by the total number of metaphase II oocytes retrieved on the basis of the recommendations of the Vienna Consensus (14).

### Statistical Analysis and Sample Size Calculation

A sample size of 90 patients was calculated on the basis of a 25% incidence of oligozoospermia (sperm concentration <15 million/ml) reported in a population of men attending an infertility center, setting type 1 and 2 errors at 0.05 and 0.20, respectively and considering as clinically relevant an increase in frequency of 15% after the vaccination (25 vs. 40% after the vaccination).

Statistical analyses were performed using Statistical Package for Social Science (SPSS) for Windows, Version 26.0 (SPSS Inc., Chicago, IL). Reference values of semen analyses were based on WHO parameters. Data are presented as number (%) mean ± Standard Deviation (SD), or median [Interquartile range–IQR] A binomial exact distribution model was used to estimate the 95% Confidence Interval (95%CI) of proportions.

## Results

One hundred and six men were ultimately included. Baseline characteristics are shown in Table 1. The median [IQR] age was 39 [36–42] years. All the subjects underwent a semen analysis before and after vaccination in the context of the infertility treatments. Eighty-two men received two vaccine doses between the two infertility treatments while twenty-two received a single dose. Frequency of the various types of vaccine received and time occurred between the vaccine exposure and the following semen analysis is reported in Table 1. Forty-five percent of the patients reported mild, self-resolving adverse events after the vaccine including pain at injection site, fever, fatigue, nausea, muscle pain, diarrhea and lymphadenopathy.

**Table 1:** Baseline characteristics of the ART population analysed.

**Characteristics**	**Number (%) or median [IQR]**
Number of cases	106
Age	39 [36–42]
**Technique**	
IUI	17 (16%)
IVF	89 (84%)
**COVID-19 Vaccines**	
Pfizer-BioNTech	73 (69%)
Moderna	20 (19%)
Oxford/AstraZeneca	10 (9%)
Johnson & Johnson's Janssen	1 (1%)
Mixed vaccines	2 (2%)
(AstraZeneca + Moderna and Pfitzer + AstraZeneca)	
Time between first vaccine dose and subsequent ART cycle (days)	75 [39–112]
Time between second dose and subsequent ART cycle^a^ (days)	59 [28–100]

a *82 patients received the vaccine second dose*.

Of the included subjects, 89 (84%) underwent two attempts of IVF while IUI procedures were performed in 17 patients. The latter population was used only for the evaluation of semen parameters.

Pairwise comparisons of fertilization rates and semen parameters before and after the exposure for the entire cohort are summarized in Table 2. Fertilization rate was similar before and after vaccination. Similarly, the various semen parameters did not change before and after the exposure. Twenty-five patients (24%) were oligozoospermic before the vaccination while 26 (25%) after the exposure (*P* = 0.87). Severe asthenozoospermia were present in 11 patients (10%) before as well as after the exposure. None of the patients was azoospermic after the vaccination nor one had a severe deterioration of the semen parameter.

**Table 2:** Fertilization rate and semen parameters as evaluated in a pairwise comparison between the first and second ART attempt, performed respectively before and after COVID-19 vaccination.

**Variables**	**Pre-vaccination**	**Post-vaccination**	***p*-value**
Volume (ml)	2.5 [1.5–3.0]	2.5 [1.8–3.0]	0.77
Concentration (M/ml)	41 [15–70]	35 [16–66]	0.82
Total N. spermatozoa (M)	94.5 [30.0–175.3]	81.3 [30.9–150.0]	0.99
Progressive motility (%)	43 [30–55]	40 [30–50]	0.14
Total motility (%)	53 [40–62]	50 [40–60]	0.21
Morphologically normal forms (%)	4 [2–6]	4 [3–6]	0.21
Total number of progressively motile spermatozoa (M)	39.0 [9.0–81.8]	32.9 [10.5–69.1]	0.77
Fertilization rate (%)	75 [50–100]	80 [50–100]	0.64

Even considering only the cohort of patients who received two doses (*n* = 82), results were similar. Median [IQR] rate of fertilization of partner's oocyte was 75 [50–100] before and 80 [50–100] after the exposure (*P* = 0.87). The median [IQR] sperm concentration/ml was 41 [14–70] before and 36 [14–66] post vaccination (*P* = 0.90) and the median [IQR] total number of spermatozoa was 86 [30–150] before and 81 [24–150] after (*P* = 0.33). Percentages of progressive motility [median (range) 41 (30–55) before and 40 (30–50) after the vaccine; *P* = 0.15] and total motility [median (range) 52 (40–62) before and 50 (40–60) after the vaccine; *P* = 0.23] did not change as well after the vaccination. Median [IQR] percentage of morphologically normal forms were 4 [2–6] before the exposure and 4 [3–6] following the vaccine, *P* = 0.09. Finally, the total number of progressively motile spermatozoa/ml was similar in the pre- and post-exposure period [median (range) 34.4 (9.0–70.2) before and 32.9 (8.9–67.9) after the vaccine; *P* = 0.55].

No difference was observed for any of the outcome considered in the pairwise comparisons before and after the vaccine exposure as divided according to the vaccine type (Supplementary Table 1).

Finally, no difference was observed for any of the outcome considered in the pairwise comparisons before and after the vaccine exposure in the subgroup of patients that reported vaccine-associated symptoms (data not shown).

## Discussion

This study was specifically designed to investigate the impact of COVID-19 vaccination on semen parameters in a cohort of infertile men belonging to couples undergoing ART programs at three tertiary referral centers in Italy. Of clinical importance, we found that COVID-19 vaccination had no impact on fertilization rate and sperm parameters. This was even true after considering different types of vaccines (mRNA or viral vector).

The study was motivated by the substantial lack of data, and the related public uncertainties, regarding the potential negative impact of COVID-19 vaccination on men's reproductive health. Particularly, little is known about the effect of COVID-19 vaccination on sperm function and quality and, because of this, one of the reported reasons for vaccine hesitancy is the potential negative effect on fertility (15).

Previous studies have demonstrated that COVID-19 infection negatively affects men's reproductive health. In terms of serum hormones, an independent association between SARS-CoV-2 infection status and secondary hypogonadism was observed, with lower testosterone levels predicting the most severe clinical outcomes (16). Furthermore, more than half of men who recovered from the disease still had circulating testosterone levels suggestive for a condition of hypogonadism after several month (17).

It is known that SARS-CoV-2 infects host cells through angiotensin-converting enzyme 2 (ACE2) receptors and that transmembrane serine protease 2 (TMPRSS2) also plays a major role in the entry of SARS-CoV-2 into the cell (18). ACE2 and TMPRSS have been shown to be highly expressed in spermatogonia and Sertoli and Leydig cells, thus suggesting that SARS-CoV-2 infection may affect the testis and lead to possible harmful effects on spermatogenesis (19). Duarte-Neto et al. described the pathological findings in testes from fatal cases of COVID-19, including the detection of viral particles and antigens and inflammatory cell subsets (20). By using post-mortem testicular samples by percutaneous puncture from 11 deceased men, Authors found decreased Leydig and Sertoli cells with reduced spermatogenesis in all cases. Immunohistochemistry detected SARS-Cov-2 antigen in Leydig cells, Sertoli cells and spermatogonia; electron microscopy detected viral particles in the cytoplasm of fibroblasts, endothelium, Sertoli and Leydig cells, spermatids, and epithelial cells of the rete testis in four cases, while RT-PCR detected SARS-CoV-2 RNA in three cases (20). Nonetheless, the presence of the virus in semen of COVID-19 patients was found to be poor. He et al. (21) analyzed the presence of SARS-CoV-2 in semen, testis, and prostatic fluid as well as the effects of COVID-19 on male reproductive function. Among the 15 semen studies in their review (290 patients considered), only one showed detection of SARS-CoV-2 in semen (6 men; 2%). Authors found that semen quality of patients with moderate infection was lower than that of patients with mild infection and healthy controls suggesting that spermatogenic dysfunction could be related to immune or inflammatory reactions (22). Similarly, Erbay et al. (22) analyzed data from 69 patients aged 20–45 years with a history of a positive test result for SARS-CoV-2 and divided the cohort into two groups according to their COVID-19 symptoms being mild or moderate. Semen samples taken before and after COVID-19 were compared between groups. Patients with moderate symptoms had worsening sperm parameters after infection compared to baseline (22). Overall, these results corroborated previous evidence suggesting that COVID-19 negatively affects sperm parameters at short-term.

Little is known about the long-term effect of COVID-19 on sperm quality. Guo et al. (23) analyzed data from 41 reproductive-aged male patients who had recovered from COVID-19 and 50 matched controls; semen parameters were considered at a median time of 56 days after hospital discharge and a second sampling was conducted for 22 patients at 84.0 (IQR: 74.0–89.0) days after hospital discharge. Compared with healthy controls, sperm concentration and progressive motility were lower in COVID-19 patients at first sampling. Of note, total sperm count, sperm concentration and motile sperm count at the second sampling significantly improved. Therefore, COVID-19 might exert adverse but potentially reversible effects on sperm quality.

Several pathophysiological mechanisms have been proposed to elucidate the negative impact of COVID-19 infection on semen quality. Oxidative stress and increased apoptosis, altered ACE2 signaling pathways and the synergistic negative contribute of air pollution were among the most frequently reported mechanism of sperm impairment by COVID-19 (24–26).

Prompted by the previous evidence of impaired reproductive function after COVID-19 infection, public concerns emerged regarding the association between SARS-CoV-2 vaccine and infertility. In this context, it was found that internet search queries in Google related to the COVID-19 vaccine and fertility significantly increased in the 48 days following Emergency Use Authorization (27). This increase in search volume suggests a desire for information about the vaccine's impact on fertility potential which could be influencing public concern and hesitancy for vaccine uptake.

Only few studies have investigated the real-life impact of COVID-19 vaccine on semen quality. Gonzalez et al. collected semen samples from 45 healthy volunteers (with no underlying fertility issues) prior to receiving the first mRNA vaccine dose and approximately 70 days after the second (5). Authors found no significant decreases in any sperm parameter after vaccination in their cohort.

In a similar study, Lifshitz et al. collected semen samples from 75 fertile men 1–2 months following the second dose of Pfizer's COVID-19 vaccine (6). The semen parameters were compared with the WHO reference ranges. Of note, only one patient (1.3%) showed sperm parameters suggestive for oligozoospermia and asthenozoospermia (6). Lastly, Orvieto et al. investigated the influence of mRNA SARS-CoV-2 vaccine on 36 couples undergoing ART treatments (28). By comparing pre/post vaccination data, Authors found no differences in the number of oocytes and mature oocytes retrieved, fertilization rate and pregnancy rate (30% per transfer). Additionally, sperm parameters from the male partner did not change after vaccination (28).

Our study corroborates theses previous findings since we showed that COVID-19 vaccination did not affect sperm quality of men undergoing ART treatments. These results were confirmed for both mRNA and viral vector vaccines. Notably, our data also indicate that COVID-19 vaccination does not impact on fertilization rate which is a critical fertility parameter because it expresses a fundamental aspect of both oocyte and sperm developmental competence. The regulatory mechanisms required for fertilization are believed to influence the development and health of the conceptus (7). Overall, our data are of utmost clinical and sociological importance since we revealed that COVID-19 vaccines are safe for men's reproductive health and they should be recommended to men seeking fertility treatment.

There are several strengths of our study. First, this is the largest multicenter study specifically designed to investigate pre vs. post COVID-19 vaccination difference in fertilization rate and sperm parameters in infertile men. Conversely, other Authors have analyzed fertile men or did not include a pre-vaccination examination, thus limiting the validity of their findings (5, 27, 28). In fact, infertile men are those who most would benefit from the lack of sperm impairment from vaccination. Second, only mRNA vaccines have been considered in previous publication (27, 28). Third, previous reports did not include the evaluation of the functional properties of the sperm. Forth, the design of the study evaluating the same cohort of men undergoing the same ART procedure before and after vaccination in a pairwise comparison allows to exclude potential biases that may derive from a case-control study.

Likewise, the study is not devoid of limitations. A selection bias might be claimed in terms of subjects who, knowing about their poor semen parameters, were particularly afraid of the vaccine consequences and refused the immunization. However, this issue is very unlikely as in Italy, about 85% of adults accepted the vaccination and this was not different in our population of patients. This rate was even higher in the Lombardy area where two of the study centers are located. As a matter of fact, we did not have a significant proportion of patients who refused to be vaccinated. On the other hand, despite being the largest series published in this topic, our results deserve external validation with an independent, larger and more diverse sample and this should be one of the recommendations of the study. In addition, results should be confirmed in long-term studies mostly because mRNA technology will be increasingly frequent in the design of new vaccines to manage various pathologies of importance in public health. Additionally, we lack data on serum hormones and patient's clinical characteristics that might affect sperm quality. Nonetheless, it is unlikely that those would change between ART cycles.

## Conclusion

Both COVID-10 vaccines, the messenger RNA (mRNA) vaccines and the vaccines utilizing a viral vector did not affect sperm quality of men undergoing ART treatments and should be considered safe for men's reproductive health.

## Data Availability Statement

The raw data supporting the conclusions of this article will be made available by the authors, without undue reservation.

## Ethics Statement

The studies involving human participants were reviewed and approved by Comitato Etico Milano Area B, N. 1083_2021; IRCCS San Raffaele Scientific Institute, ID BC-GINEOS, and Ospedale Careggi, Firenze N. 18111, 20/05/2021. The patients/participants provided their written informed consent to participate in this study.

## Author Contributions

MR and PV conceived the study. VB, GF, CD, FP, and GCC collected the data. LP and MR analyzed the data. PV, LB, and ES wrote the first draft. MEC and EP revised the manuscript. All authors discussed the results and contributed to the final manuscript.

## Conflict of Interest

The authors declare that the research was conducted in the absence of any commercial or financial relationships that could be construed as a potential conflict of interest.

## Publisher's Note

All claims expressed in this article are solely those of the authors and do not necessarily represent those of their affiliated organizations, or those of the publisher, the editors and the reviewers. Any product that may be evaluated in this article, or claim that may be made by its manufacturer, is not guaranteed or endorsed by the publisher.
