# Supplementary material for: COVID-19 Vaccination Does Not Affect Reproductive Health Parameters in Men
Source: Front Public Health. 2022 Feb 2;10:839967. doi: 10.3389/fpubh.2022.839967 (PMC8847439; doi:10.3389/fpubh.2022.839967)
Supplement: Supplementary file 1 [file Table_1.DOCX]

**Supplementary Table 1.** Fertilization rate and semen parameters as evaluated in a pairwise comparison between the first and second ART attempt, performed respectively before and after COVID-19 vaccination and according to the different COVID-19 Vaccines

|  |  |  |  |  |
| --- | --- | --- | --- | --- |
| Variables | Pre-vaccination | Post-vaccination | p |  |
|  |  |  |  |  |
|  |  |  |  |  |
| mRNA vaccine (n=93) |  |  |  |  |
| Volume (ml) | 2.4 [1.5 - 3.0] | 2.5 [1.8 - 3.0] | 0.44 |  |
| Concentration (M/ml) | 41 [12 - 70] | 35 [14 - 62] | 0.54 |  |
| Total N. spermatozoa (M) | 90 [30 - 175] | 76 [31 - 150] | 0.81 |  |
| Progressive motility (%) | 43 [30 - 55] | 40 [30 - 50] | 0.15 |  |
| Total motility (%) | 53 [40 - 63] | 50 [40 - 60] | 0.21 |  |
| Morphologically normal forms (%) | 4 [2 - 5] | 4 [3 - 5] | 0.45 |  |
| Total number of progressively motile spermatozoa (M) | 40.0 [9.0 - 80.4] | 28.3 [11.0 - 65.8] | 0.73 |  |
| Fertilisation rate (%) | 75 [50 - 100] | 80 [50 - 100] | 0.87 |  |
| Viral vectors (n=11) |  |  |  |  |
| Volume (ml) | 2.6 [1.5 - 3.5] | 2.5 [1.4 - 3.0] | 0.59 |  |
| Concentration (M/ml) | 25 [22 - 80] | 28 [20 - 82] | 0.25 |  |
| Total N. spermatozoa (M) | 75 [46 - 196] | 104 [20 - 315] | 0.37 |  |
| Progressive motility (%) | 40 [10 - 50] | 35 [25 - 45] | 0.86 |  |
| Total motility (%) | 50 [15 - 60] | 50 [30 - 60] | 0.67 |  |
| Morphologically normal forms (%) | 4 [2 - 7] | 4 [3 - 7] | 0.18 |  |
| Total number of progressively motile spermatozoa (M) | 20.8 [7.5 - 70.0] | 39.0 [7.0 - 88.5] | 0.42 |  |
| Fertilisation rate (%) | 72 [43 - 98] | 84 [69 - 100] | 0.48 |  |
|  |  |  |  |  |
|  |  |  |  |  |
